# Photodynamic diagnosis of ovarian cancer using hexaminolaevulinate: a preclinical study

**DOI:** 10.1038/sj.bjc.6600958

**Published:** 2003-05-27

**Authors:** F Lüdicke, T Gabrecht, N Lange, G Wagnières, H van den Bergh, L Berclaz, A L Major

**Affiliations:** 1 Fondation pour Recherches Médicales, University of Geneva, 64 Avenue de la Roseraie, 1211 Geneva, Switzerland; 2 Institute of Environmental Engineering, Swiss Federal Institute of Technology (EPFL) Lausanne, Switzerland; 3 Department of Obstetrics and Gynaecology, University Hospital Geneva, Switzerland

**Keywords:** photodynamic diagnosis, ovarian cancer

## Abstract

The unfailing detection of micrometastases during surgery of patients suffering from ovarian cancer is mandatory for the optimal management of this disease. Thus, the present study aimed at determining the feasibility of detecting micrometastases in an ovarian cancer model using the intraperitoneal administration of the photosensitiser precursor hexaminolaevulinate (HAL). For this purpose, HAL was applied intraperitoneally at different concentrations (4–12 mM) to immunocompetent Fischer 344 rats bearing a syngeneic epithelial ovarian carcinoma. The tumours were visualised laparoscopically using both white and blue light (D-light, Karl Storz, Tuttlingen, Germany), and the number of peritoneal micrometastases detected through HAL-induced photodiagnosis (PD) was compared to standard white light visualisation. Fluorescence spectra were recorded with an optical fibre-based spectrofluorometer and the fluorescence intensities were compared to the protoporphyrin IX (PpIX) fluorescence induced by 5-aminolevulinic acid under similar conditions. The number of metastases detected by the PD blue light mode was higher than when using standard white light abdominal inspection for all applied concentrations. Twice as many cancer lesions were detected by fluorescence than by white light inspection. The hexyl-ester derivative produced higher PpIX fluorescence than its parent substance aminolevulinic acid at the same concentration and application time. Fluorescence contrast between healthy and cancerous tissue was excellent for both compounds. To overcome poor diagnostic efficiency and to detect peritoneal ovarian carcinoma foci in the large surface area of the human peritoneal cavity, HAL fluorescence-based visualisation techniques may acquire importance in future and lead to a more correct staging of early ovarian cancer.

Only ovarian cancer patients who have undergone a thorough surgical staging procedure, with early-stage and well or moderately differentiated tumours, may be considered as cured by surgery alone, and therefore do not require adjuvant therapy (Trimbos *et al*, 1991). Occult extra-ovarian metastases are found in about 15–20% of patients initially diagnosed as early ovarian cancer confined to the ovary. The confirmed absence of such micrometatstatic lesions in the peritoneal cavity does have impact on the decision-making process of the physician with respect to the treatment regimen as well as on patient's quality of life.

Statistically, these occult lesions are found in the diaphragm (7%), the omentum (5%), peritoneal biopsy specimens (10%), and peritoneal cytology (20%) (Leblanc *et al*, 2000).

The magnitude of the problem becomes even more apparent following optimally performed re-staging operations. Up to 30% of patients with presumed early-stage ovarian cancer will be upstaged if re-explored, and approximately two-thirds of these patients will have stage III disease (Bagley Jr *et al*, 1973; Piver, 1982; Young *et al*, 1983; Helewa *et al*, 1986; Buchsbaum *et al*, 1989; Soper *et al*, 1992). Despite attempts at improving staging through computed tomography (CT), magnetic resonance imaging (MRI), and other established imaging diagnostic tools, the detection of residual micrometastatic disease remains a challenge to the gynaecological oncologist and collaborating disciplines.

To overcome poor diagnostic efficiency in the large surface area of the human peritoneal cavity, fluorescence-based visualisation techniques might acquire greater importance in the future.

Fluorescence photodetection (PD) takes advantage of the optical properties of the tissues, either inherent (autofluorescence) or induced by exogenously administered photoactive compounds. The fact that cancerous tissue often shows differences not only in its biological behaviour, but also in the resulting optical characteristics has allowed PD of malignant disease in fields such as urology, (Jichlinski *et al*, 1997a,1997b; Kriegmair *et al*, 1999) dermatology (Orenstein *et al*, 1997) and pulmology (Monnier Ph *et al*, 1990).

Although not a photoactive compound itself, exogenously administered 5-aminolaevulinic acid (ALA), the first committed intermediate in the haeme biosynthetic pathway, results in a temporary accumulation of fluorescing porphyrin precursors, particularly protoporphyrin IX (PpIX). Administration of exogenous ALA bypasses the negative feedback control and induces the preferential accumulation of PpIX in malignant cells of epithelial origin (for a review, see Peng *et al*, 1997) The fluorescence of PpIX can easily be used for diagnostic purposes when excited by light with in the blue range of the visible spectrum (Wagnieres *et al*, 1998). In many medical areas, ALA-induced PD has replaced the photosensitiser compounds used previously, because of its simple application scheme (systemic or topical), almost complete absence of side effects, short-lasting phototoxicity, and good discrimination between malignant and nonmalignant tissue (Peng *et al*, 1997).

We were previously able to show in a preclinical and clinical setting that ALA-induced PD can successfully make visible ovarian cancer micrometastases in the peritoneal cavity (Major *et al*, 1997,2002a,2002b; Hornung *et al*, 1998). While ALA-induced PD has proven its feasibility in detecting ovarian cancer micrometastases, the pharmacological properties of ALA make it suboptimal when applied topically or, as in our research, intraperitoneally in ovarian cancer patients. 5-Aminolaevulinic acid is a hydrophilic molecule, and its penetration across biological membranes is partially compromised. 5-Aminolaevulinic acid-induced PpIX formation after topical application shows considerable heterogeneity and the depth of penetration is limited (Lange *et al*, 1999,^2001^). More lipophilic derivatives of ALA are currently under clinical assessment, in attempts to enhance ALA's poor bioavailability. It has been shown that the hexyl-ester derivative of ALA (hexaminolaevulinate (HLA)) represents a good compromise between aqueous solubility and lipophilicity.

Our aim was to determine the feasibility of detecting micrometastases in an ovarian cancer model using the intraperitoneal administration of the photosensitiser precursor HAL. Additionally, we aimed to quantify the peritoneal micrometastases through HAL-induced PD, and to compare the number of detected lesions with the one obtained through standard white light visualisation.

## MATERIALS AND METHODS

Female Fischer (F-344) rats (120–160 g) were housed in a pathogen-free animal facility at the ‘Fondation pour la recherche médicale’, Geneva. They were given unlimited access to commercial basal diet and water. The experimental protocol for the use of animals for these studies was approved by the Institutional Ethics Review Board and the local veterinary office. The study was carried out according to the UKCCCR guidelines for the welfare of animals in experimental neoplasia (Workman *et al*, 1998). In particular, it should be noted that the experimental system was set up so as to detect ovarian cancer metastases at a very early stage of the disease when implants are not or only difficult to visualise with the naked eye. In our system, this was the case after 5 weeks of tumour induction.

The NuTu-19 cell line is a poorly differentiated Fischer 344 rat-derived epithelial ovarian cancer cell line (Rose *et al*, 1996). Cells were cultured in DMEM medium (Gibco Life Technologies, Carlsbad, USAPlease provide the location of the supplier.) enriched with 10% foetal calf serum (Gibco Life Technologies), penicillin 25 IE ml^−1^, streptomycin 25 mg ml^−1^, and incubated under standardised conditions (37°C, 7% carbon dioxide, 100% humidity). One million cells per animal were harvested with 0.25% trypsin (Gibco Life Technologies), washed with phosphate-buffered saline (PBS) (Gibco Life Technologies) and injected intraperitoneally.

At 5 weeks after tumour induction, crystallised ALA hydrochloride (Merck, Darmstadt, Germany) was diluted in PBS and titrated with NaOH to pH 7.4. The synthesis of HAL is described elsewhere (Kloek *et al*, 1998; Lange *et al*, 1999). In all, 2 ml of solution (4–12 mM HAL in PBS titrated with HCl to pH 7.4, 8 mM ALA) was injected intraperitoneally into each rat.

For PD, the D-Light system (Storz™) was used. A 300 W xenon short-arc lamp emitted blue light in the range from 375 to 440 nm. A foot pedal made it possible to switch between blue–violet fluorescence excitation light and white light. In the endoscopic eyepiece, a yellow long-pass filter reduces the reflected blue light, intensifying the contrast between the red fluorescent and nonfluorescent areas of the healthy surrounding tissue. Tumours and premalignant lesions fluoresces bright red in the violet excitation light.

Point spectroscopy is a highly sensitive method for measuring fluorescence signals, making it a useful tool for *in vivo* pharmacokinetic measurements. In this study, fluorescence emission spectra were recorded with an optical fibre-based spectrofluorometer based on a Peltier-cooled CCD coupled to a spectrograph (Cromex 250, SI Instruments, Germany). Excitation light (*λ*_ex_=405 nm) from a 75 W high-pressure xenon lamp (UXL-75 XE, Ushio Inc., Japan) was spectrally resolved by a quarter metre monochromator (Chromex 250, SI Instruments, Germany) with a bandwidth of 5 nm and an excitation filter, SCHOTT BG3 (Schott AG, Mainz, Germany), mounted on a filter wheel. A stepper motor (SMC 100, Princeton Instruments Inc., USA) controlled this excitation filter wheel, which was equipped with different low-pass filters to purify the excitation light. Excitation energy measured at the distal end of the fibre tip was determined with a calibrated power-metre (Optical Power Meter 840, Newport, USA). A filter setup allows the acquisition of fluorescence emission spectra between 455 and 900 nm. The setup and data acquisition were controlled by a 486 personal computer using CSMA software (SI Instruments GmbH, Germany). An aqueous solution of rhodamine B (*c*=1 × 10^−6^ mol l^−1^) in a 10 mm quartz cuvette was used as a reference. Emission spectra of the reference were recorded before and after each measurement. All measurements were normalised to the peak value of the reference to give comparable results corrected for day-to-day fluctuations in the excitation light energy or detection pathway alignment. Point fluorescence measurements of cancerous and normal tissue were performed with a 500 *μ*m fibre probe. Each point measurement was repeated three times. The peritoneal autofluorescence spectrum of a rat without any medication was used as reference spectrum, and all data of the spectrofluorometer presented in this work are the measured fluorescence values after subtraction of the autofluorescence.

A total of 11 photosensitised rats were killed 2 h after instillation of ALA or HAL. A midline incision from the xyphoid process to the symphysis pubis was made and the viscera were explored. To avoid bleaching of the photosensitiser, all procedures were carried out under dimmed room light.

The number of lesions in each photosensitised animal was counted independently by two investigators, one performing examination under white light and the other using fluorescence diagnosis. Several biopsies were taken from fluorescent sites.

## RESULTS

Metastases were most frequently found on the caudal side of the diaphragm, the peritoneum, and less frequently on the omentum and intestine. All biopsies taken from lesions seen by normal inspection or detected through PD were histopathologically proven cancer.

The number of metastases detected by the PD blue light mode was significantly higher (*χ*^2^ test, *P*<0.01) than when using standard white light abdominal inspection for all applied concentrations (Table 1

**Table 1:** Numbers of metastases detected by white light and blue light detection using HAL (4–12 mM) and ALA (8 mM)

**Concentration (mM) (number of animals)**	**Precursor**	**Time after injection (h)**	**White light**	**Blue light**	**Ratio**
4 (3)	HAL	2.5	9	23	2.6
8 (4)	HAL	2.0	70	123	1.8
12 (2)	HAL	2.0	3	8	2.7
8 (2)	ALA	2.0	10	16	1.6

). About twice as many cancer lesions were detected by PpIX fluorescence than by white light inspection. Figure 1

**Figure 1:**
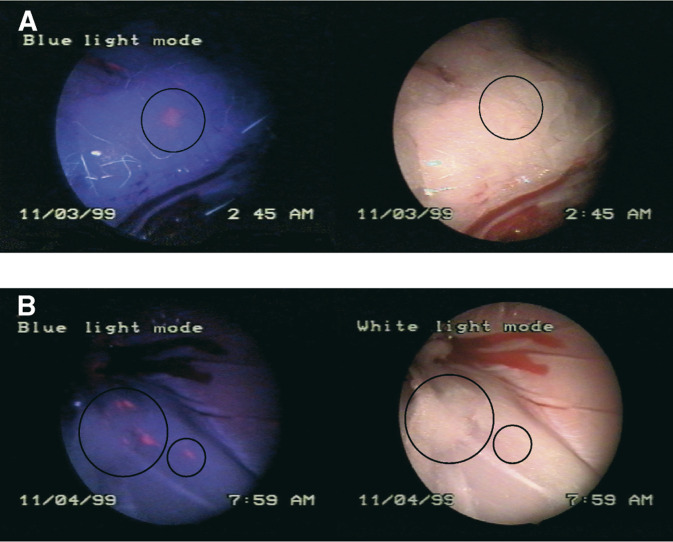
Peritoneal metastases in blue and white light mode. Image (**A**) shows a lesion that is only visible in the blue light mode, but not by white light (position marked by a circle) (8 mM HAL after 2 h). Image (**B**) shows three lesions visible by both blue and white light (big circle) and one only detectable by fluorescence (small circle) (8 mM HAL after 2 h).

shows a sequence of white light and blue light images of small cancerous metastases through PD 2 h following injection of HAL (8 mM), which would have been missed by standard white light diagnosis.

**Figure 2:**
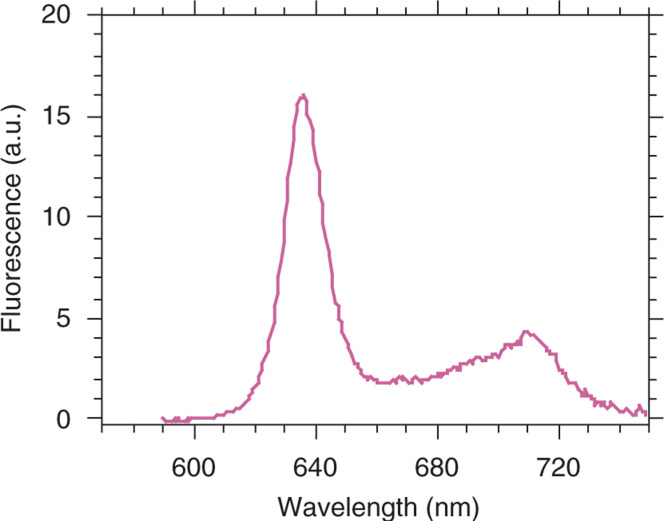
Typical PpIX fluorescence spectrum of peritoneal ovarian cancer metastasis 2 h after i.p. administration of 8 mM HAL.Delete ‘B’ in figure?

A typical fluorescence spectrum registered by point spectrofluorometric measurement of a metastasis is shown in Figure 2

. It depicts the characteristic fluorescence emission spectrum of PpIX and was the same independently on the used concentration or precursor.

**Figure 3:**
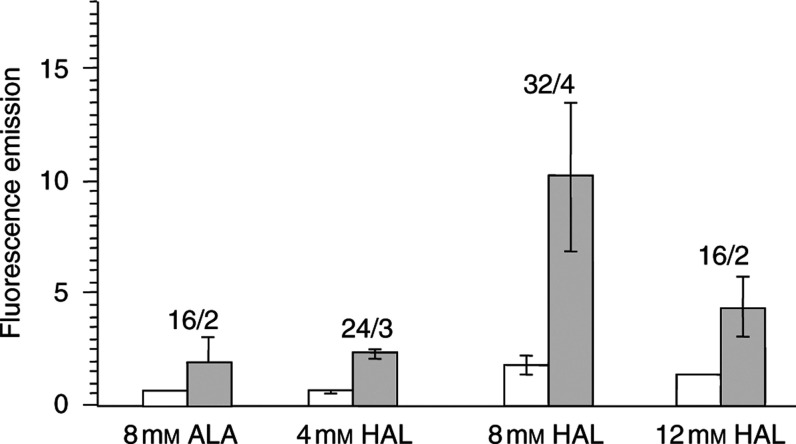
Mean fluorescence emission of healthy peritoneal (empty box) and cancerous (grey box) tissue. Four different sites for normal tissue and four sites of cancerous tissue were measured in each rat. In all, 8 mM ALA and 4–12 mM HAL were applied and fluorescence was measured 2 h after injection (number of measurements/number of animals; bar=s.d.).

In total, we measured 44 tumour sites and 44 normal tissue sites in 11 rats. The mean values for each condition for tumour and normal tissue are shown in Figure 3

. Tumour to normal tissue ratios for the fluorescence intensities ranged from 2.7 (4 mM HAL) to 10 for HAL at 8 mM. The mean fluorescence intensities were significantly different (Mann–Whitney *U*-test, *P*<0.01) between normal and cancerous tissue for ALA and HAL. Increasing the dosage of HAL from 8 to 12 mM did not further increase the fluorescence harvest.

At 8 mM concentration, the hexyl-ester derivative produced significantly higher PpIX fluorescence as compared to ALA (Figure 3); however, visually the fluorescence contrast between healthy and cancerous tissue was excellent for both the compounds.

## DISCUSSION

Owing to its good tumour selectivity and excellent clinical tolerance, the photoactive precursor ALA is increasingly used as photosensitiser in photodynamic therapy and fluorescence photodetection. 5-Aminolaevulinic acid can be applied topically (creams, instillation) or systemically (oral, inhalation) (Peng *et al*, 1997). 5-Aminolaevulinic acid-induced PpIX appears to be cleared from the body within 24 h of induction, whether the route is systemic or topical (Webber *et al*, 1997). Systemic administration of doses >30 mg kg^−1^ resulted in a decrease of systolic blood pressure with a median of 80 mmHg 6–7 h later. Lower doses did not affect the haemodynamic variables (Heyerdahl *et al*, 1997; Webber *et al*, 1997). To date, no side effects have been reported following topical application of ALA. Similarly, installation of up to 16 mM HAL into the bladder did not result in any side effects (Lange *et al*, 1999).

Gahlen *et al* (2001),(2002) have shown that local, intraperitoneal photosensitisation with ALA is a more reliable and effective method than systemic photosensitisation with ALA for detection of small or occult peritoneal tumours. More lipophilic derivatives of ALA, such as HAL, that have better bioavailability than ALA should therefore show good performance in the detection of micrometastases when given intraperitoneally.

To test HAL-induced fluorescence *in vivo*, we utilised the NuTu-19 epithelial ovarian cancer animal model. This model closely simulates clinical human ovarian cancer in terms of (a) method of intraperitoneal spread, (b) formation of malignant ascites, and (c) propensity for local metastases and organ invasion (omentum, peritoneum, liver, spleen, bowel). Our previous experiments with ALA tested time intervals between 0.5 and 4 h at concentrations between 4 and 20 mM, and showed best discrimination between healthy and cancerous tissue when 8 mM ALA was applied for 2 h (Major *et al*, 1997,2002a,2002b). We therefore compared ALA with HAL at this dosage and time interval. At 8 mM, intraperitoneal application of HAL results in twice as much PpIX formation than the same amount of ALA. These data confirm earlier reports of better fluorescence yield of HAL when compared to ALA *in vitro* (Marti *et al*, 1999) and *in vivo* (Lange *et al*, 1999), and may permit, in clinical use, shorter drug application time while retaining tumour selectivity.

In our model, both HAL and its parent molecule effectively detected small cancerous lesions. Half of these small tumours would have been missed by normal inspection of the abdominal cavity. We have demonstrated that HAL-induced fluorescence is a convenient tool that improves the contrast of fluorescing metastases against healthy tissue and allows the detection of cancer lesions that would not have been discovered by normal inspection. However, from our experiments, we cannot exclude that the conditions of the experimental model used may not have been optimal for HAL, and that varying experimental conditions, such as incubation time with HAL or its delivery vehicle, might further improve the selectivity of fluorescence localisation.

Photodiagnostic techniques such as HAL detection of occult ovarian cancer tumours provide a platform technology that permits minimally intrusive investigation, and could allow detection by endoscopy and eventually elimination of ovarian cancer cells at their earliest stages. Moreover, detection, diagnosis, and treatment could be closely coupled, enabling effective administration in a single, seamless process. To start with, laparoscopic staging of early ovarian cancer would benefit from this simple and straightforward method of photodetection. Hexaminolaevulinate has greater potency to induce fluorescence in cancerous lesions, and with the acquisition of further data on safety in human, it may replace ALA in topical applications.
